# Effects of Chronic Caffeine Consumption on Synaptic Function, Metabolism and Adenosine Modulation in Different Brain Areas

**DOI:** 10.3390/biom13010106

**Published:** 2023-01-04

**Authors:** Cátia R. Lopes, Andreia Oliveira, Ingride Gaspar, Matilde S. Rodrigues, Joana Santos, Eszter Szabó, Henrique B. Silva, Ângelo R. Tomé, Paula M. Canas, Paula Agostinho, Rui A. Carvalho, Rodrigo A. Cunha, Ana Patrícia Simões, João Pedro Lopes, Samira G. Ferreira

**Affiliations:** 1 CNC—Center for Neuroscience and Cell Biology, University of Coimbra, 3004-504 Coimbra, Portugal; 2 Department of Life Sciences, Faculty of Sciences and Technology, University of Coimbra, 3000-456 Coimbra, Portugal; 3 Faculty of Medicine, University of Coimbra, 3004-504 Coimbra, Portugal

**Keywords:** caffeine, adenosine receptors, synapse, metabolism, LTP

## Abstract

Adenosine receptors mainly control synaptic function, and excessive activation of adenosine receptors may worsen the onset of many neurological disorders. Accordingly, the regular intake of moderate doses of caffeine antagonizes adenosine receptors and affords robust neuroprotection. Although caffeine intake alters brain functional connectivity and multi-omics analyses indicate that caffeine intake modifies synaptic and metabolic processes, it is unclear how caffeine intake affects behavior, synaptic plasticity and its modulation by adenosine. We now report that male mice drinking caffeinated water (0.3 g/L) for 2 weeks were behaviorally indistinguishable (locomotion, mood, memory) from control mice (drinking water) and displayed superimposable synaptic plasticity (long-term potentiation) in different brain areas (hippocampus, prefrontal cortex, amygdala). Moreover, there was a general preservation of the efficiency of adenosine A_1_ and A_2A_ receptors to control synaptic transmission and plasticity, although there was a tendency for lower levels of endogenous adenosine ensuring A_1_ receptor-mediated inhibition. In spite of similar behavioral and neurophysiological function, caffeine intake increased the energy charge and redox state of cortical synaptosomes. This increased metabolic competence likely involved a putative increase in the glycolytic rate in synapses and a prospective greater astrocyte–synapse lactate shuttling. It was concluded that caffeine intake does not trigger evident alterations of behavior or of synaptic plasticity but increases the metabolic competence of synapses, which might be related with the previously described better ability of animals consuming caffeine to cope with deleterious stimuli triggering brain dysfunction.

## 1. Introduction

The regular intake of moderate doses of coffee is increasingly recognized to decrease the incidence of numerous chronic diseases, namely, upon aging (e.g., [1,2]). Notably, caffeine, the most widely consumed psychoactive drug worldwide, recapitulates the reduction in overall mortality (e.g., [3,4]), which supports the contention that caffeine might be a prime effector of the impact of coffee on human health, although other coffee constituents such as chlorogenic acids might also contribute to the health benefits afforded by the regular consumption of moderate doses of coffee (e.g., [5]). Caffeine mostly acts through the antagonism of adenosine receptors [6]. In particular, the impact of caffeine on information flow in brain circuits is fully accounted by the combined antagonism of adenosine A_1_ receptors (A_1_R) controlling basal synaptic transmission and of adenosine A_2A_ receptors (A_2A_R) controlling synaptic plasticity [7]. As occurs for A_2A_R antagonists, moderate doses of caffeine afford a robust neuroprotection in different animal models of neuropsychiatric diseases, ranging from Alzheimer’s disease [8,9], Parkinson’s disease [10], ischemia [11,12], epilepsy/convulsions [13,14,15], traumatic brain injury [16,17], diabetic encephalopathy [18,19], spinocerebellar ataxia type 3 [20,21], multiple sclerosis [22,23], attention deficit and hyperactivity disorder [24,25] or depressive-like mood dysfunction [26,27,28].

Importantly, the neuroprotective effects of caffeine require its prolonged intake whereas the acute administration of caffeine to naïve animals often has effects opposite to these afforded by a continuous intake of caffeine (reviewed in [29,30]): the acute administration of caffeine aggravates convulsions through inhibitory A_1_R [31,32], as well as brain traumatic or ischemic damage [12,33,34]; in contrast, a regular exposure to caffeine has a neuroprotective effect through the attenuation of facilitatory A_2A_R signalling [9,15,27,35]. Thus, it appears that repeated exposure to caffeine might induce a form of brain preconditioning, with reported alterations of brain functional connectivity [36,37,38], brain metabolism (e.g., [18,39,40,41]) and levels of adenosine [42], as well as of A_1_R and A_2A_R in the adult brain (e.g., [43,44]). 

These long-term adaptive effects caused by long-term exposure to caffeine were assessed in whole brain preparations, whereas the onset of brain dysfunction involves synaptic dysfunction (reviewed in [45,46,47,48]). Accordingly, the targets of caffeine in the brain (A_1_R and A_2A_R; see [49]) are mostly located in synapses [50,51], and neuronal adenosine receptors are involved in brain damage in noxious conditions [27,52,53] through a control of the dysfunction and damage of synapses (reviewed in [30]), which only represent circa 1–2% of the brain parenchyma [54]. However, it is unknown if the prolonged exposure to caffeine alters the efficiency of the adenosine neuromodulation system in excitatory synapses of the brain.

Thus, we investigated in adult mice how the prolonged exposure to caffeine affects synaptic transmission and the function of adenosine receptors in synapses of different brain regions, and we also tested if there were caffeine-induced alterations of primary metabolism, which are known to impact on synaptic function and susceptibility to noxious brain stimuli (reviewed in [55,56]). 

## 2. Results

### 2.1. Altered Caffeine Levels upon Regular Intake of Caffeine over Time

**Figure 1:**
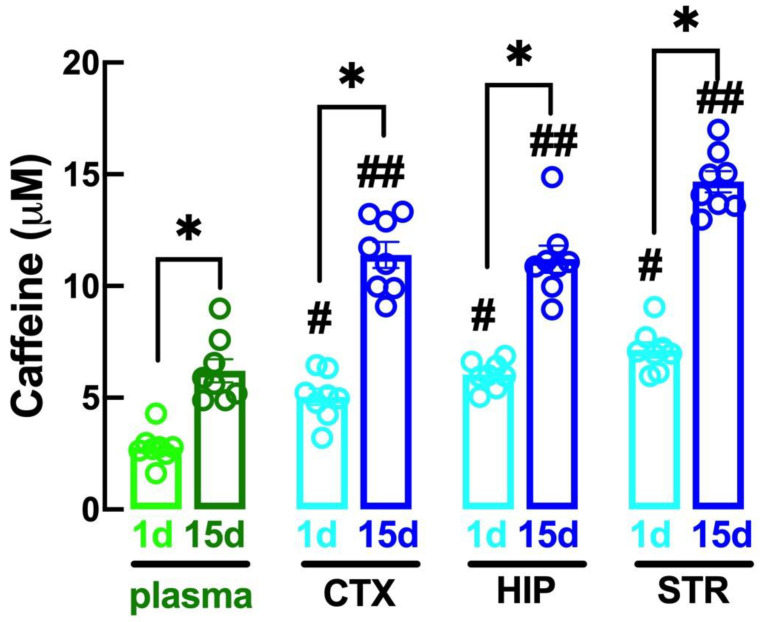
The brain levels of caffeine are higher than in the plasma and increase from acute to chronic intake of caffeine. Caffeine was quantified in the plasma and in extracts from different brain areas (CTX, cerebral cortex; HIP, hippocampus; STR, striatum) collected from adult mice after 1 day (lighter colors) or after 15 days (darker colors) of consumption of caffeinated water (0.3 g/L). The bars are mean ± SEM of 8 experiments (number of different animals tested). * *p* < 0.05 between indicated bars, # *p* < 0.05 compared with values in the plasma at day 1, ## *p* < 0.05 compared with values in the plasma at day 15, all using Student’s *t* tests with Welsh correction.

We first assessed the levels of caffeine in the plasma and in the brain parenchyma of adult mice after an acute and upon regular intake of caffeinated water (0.3 g/L). As shown in Figure 1, the plasma levels of caffeine after 24 h of access to caffeinated water were 2.47 ± 0.30 μM in the plasma (n = 8), and ranged from 4.92 ± 0.67 μM in the cerebral cortex, 6.08 ± 0.30 μM in the hippocampus and 7.06 ± 0.71 μM in the striatum (n = 8); this shows that caffeine levels were higher in the brain parenchyma than in the plasma, as expected from the higher lipophilicity of the brain parenchyma compared with the plasma. Notoriously, the levels of caffeine were higher in all compartments after 15 days of regular intake of caffeine, reaching levels of 4.89 ± 0.57 μM in the plasma (n = 8), and higher levels (*p* < 0.05, n = 8; Student’s *t* test) in the cerebral cortex (11.75 ± 0.79 μM), hippocampus (11.64 ± 1.24 μM) and striatum (14.41 ± 0.71 μM) (Figure 1).

### 2.2. Lack of Evident Behavioral Modifications upon Caffeine Intake

**Figure 2:**
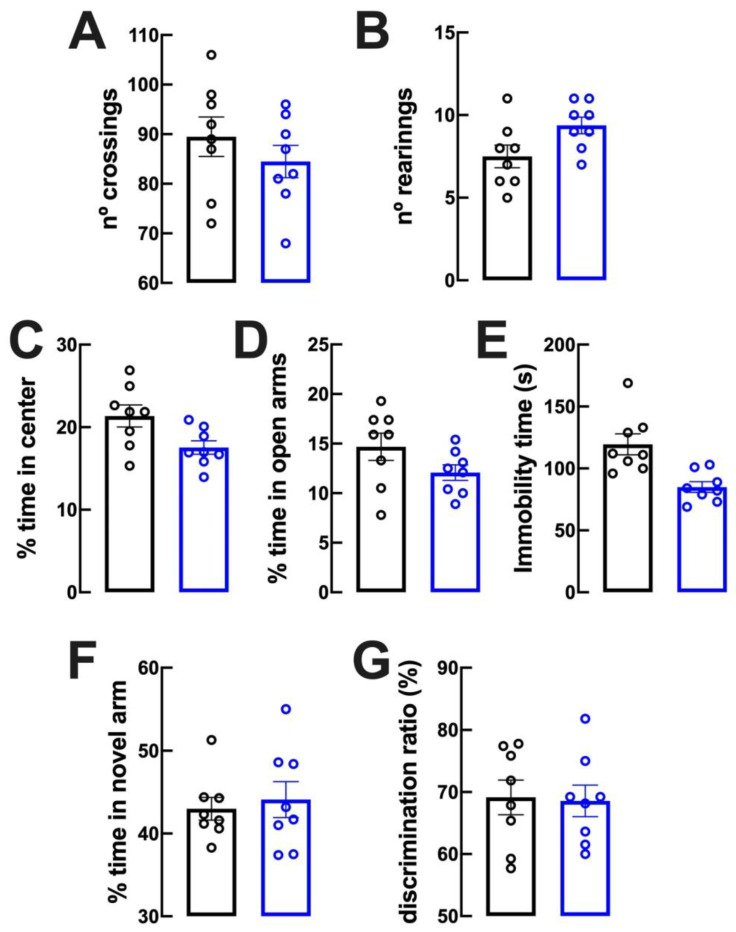
The chronic intake of caffeine does not modify locomotor, mood or memory behavior. Male adult mice (10 weeks old) consumed either caffeine (0.3 g/L, blue bars) or water (control, black bars) for 15 days before beginning behavioral analysis. There was no evident modification of either locomotor activity as evaluated by the number of crossings in the open field (**A**), sensorimotor integration as evaluated by the number of rearings in the open field (**B**), anxiety-like behavior as evaluated in the open field (**C**) and in the elevated-plus maze (**D**) tests, helpless-like behavior as evaluated by the forced-swimming test (**E**) and spatial reference memory performance as evaluated by a modified Y maze test (**F**) and an object-displacement test (**G**) between the two groups. Data are mean ± SEM; n = 8 rats per group.

We then characterized some behavioral features of mice regularly consuming caffeinated water. After 13–18 days of caffeine intake, mice displayed no modification of locomotion (number of crossings during 10 min in an open field, Figure 2A) nor of sensorimotor integration (number of rearing events during 10 min in an open field, Figure 2B) nor of anxiety-like behavior (percentage of time spent in the center of an open field during 10 min, Figure 2C). The lack of an altered anxiety-like behavior was further confirmed in an elevated plus maze, where mice drinking caffeine or water (as control) spent a similar percentage of time in the open arms (Figure 2D). Other mood-related behaviors were also unaltered upon regular intake of caffeine, as assessed by the similar immobility time accounted for mice drinking caffeine or water (as control) in a forced swimming test (Figure 2E). We next probed for possible alterations of reference memory performance upon regular consumption of caffeine: we observed no alterations in the percentage of time spent in the novel arm in a modified Y-maze (Figure 2F), as well as no alterations in the discrimination index in an object displacement test (Figure 2G) between mice drinking caffeinated water or water (n = 8 in all behavioral tests).

### 2.3. Impact of Caffeine Intake on Adenosine Modulation of Synaptic Transmission and Plasticity in the Hippocampus

**Figure 3:**
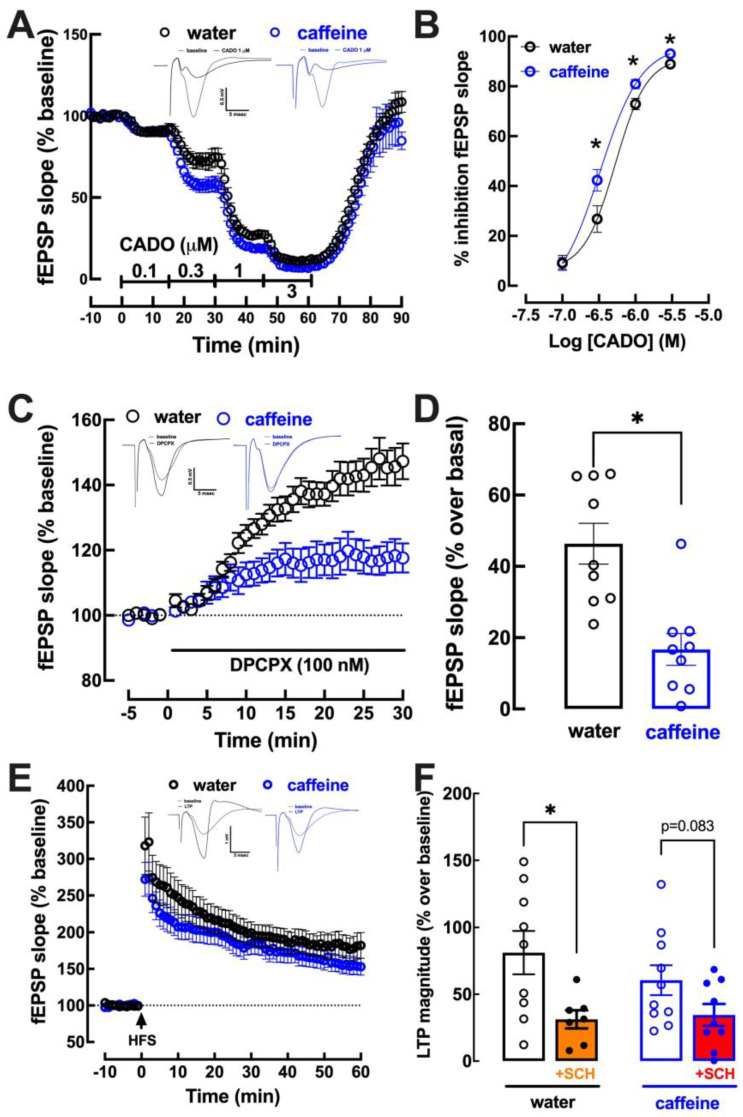
Impact of caffeine intake on the adenosine modulation of synaptic transmission and plasticity in the hippocampus. Male adult mice (10 weeks old) consumed either caffeine (0.3 g/L, blue symbols and bars) or water (control, black symbols and bars) for 19–21 days before sacrifice. Synaptic transmission was measured in Schaffer fibers-CA1 pyramid synapses by measuring the slope of field excitatory postsynaptic potentials (fEPSP), as shown in the inserts in (**A**). The activation of adenosine A_1_ receptor by 2-chloroadenosine (CADO) caused a concentration-dependent inhibition with greater efficiency upon caffeine treatment (**A**,**B**). The levels of endogenous adenosine within hippocampal excitatory synapses were lower upon caffeine treatment, as indirectly assessed by the disinhibition of synaptic transmission by the A_1_ receptor antagonist DPCPX (**C**,**D**). Long-term potentiation (LTP), triggered by a high-frequency train (HFS, 100 Hz for 1 s), displayed a similar magnitude (quantified as the percentage change between the average slope of the 10 averaged potentials taken between 50 and 60 min after LTP induction in relation to the average slope of the fEPSP measured during the 10 min that preceded LTP induction) in caffeine- and water-consuming mice (**E**,**F**) and the selective antagonist of adenosine A_2A_ receptors, SCH58261 (SCH), caused a significant reduction in LTP in control mice but not in caffeine-treated mice (**F**). Data are mean ± SEM of 7–10 experiments (number of different animals tested); * *p* < 0.05 using a Student’s *t* test with Welsh correction.

Since caffeine mostly acts through the antagonism of adenosine receptors involved in the control of synaptic transmission and plasticity [7], we next investigated if the regular consumption of caffeine modified the control of synaptic transmission and plasticity that is modulated in different brain regions by adenosine A_1_ receptors (A_1_R) and adenosine A_2A_ receptors (A_2A_R), respectively [7,57,58,59]. In Schaffer fibers-CA1 pyramid synapses of mouse hippocampal slices, the activation of A_1_R by 2-chloroadenosine [60] caused a larger inhibition of synaptic transmission in mice drinking caffeine than in control mice (Figure 3A,B). Additionally, the tonic activation of A_1_R by endogenous adenosine was lower in mice drinking caffeine than in control mice, as concluded from the lower (*p* < 0.05; Student’s *t* test) disinhibition of synaptic transmission by a supramaximal concentration of the selective A_1_R antagonist DPCPX (100 nM; [61]) in mice drinking caffeine than in control mice (Figure 3C,D). Finally, there was no significant difference (*p* = 0.315; Student’s *t* test) of the magnitude of long-term potentiation (LTP) in hippocampal slices from mice drinking caffeine (60.6 ± 11.2% over baseline, n = 9) or water (81.1 ± 16.3% over baseline, n = 10) (Figure 3E,F). Moreover, there was a tendency for a decreased impact of A_2A_R in the control of synaptic plasticity, since a supramaximal concentration of the selective A_2A_R antagonist SCH58261 (50 nM; [62]) significantly (*p* < 0.05; Student’s *t* test) decreased LTP magnitude in control mice, whereas there was only a tendency (*p* = 0.083; Student’s *t* test) for an inhibition of LTP magnitude in mice drinking caffeine (Figure 3F).

### 2.4. Impact of Caffeine Intake on Adenosine Modulation of Synaptic Transmission and Plasticity in the Prefrontal Cortex

**Figure 4:**
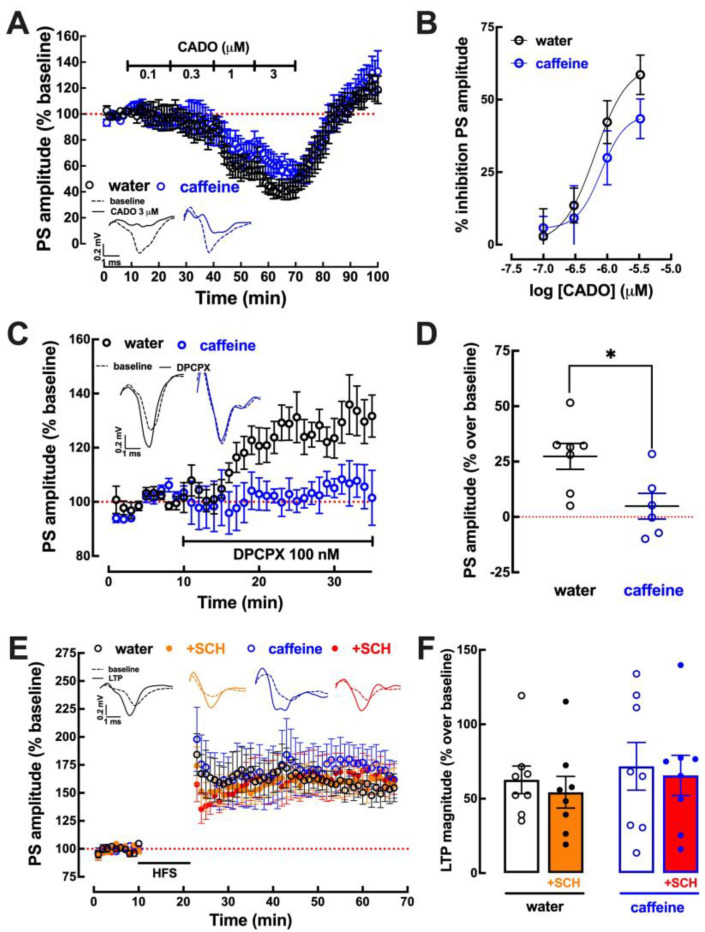
Impact of caffeine intake on the adenosine modulation of synaptic transmission and plasticity in the prefrontal cortex. Male adult mice (10 weeks old) consumed either caffeine (0.3 g/L, blue symbols and bars) or water (control, black symbols and bars) for 19–21 days before sacrifice. Synaptic transmission between pyramidal neurons in layer II/III and layer V was measured as the amplitude of the population spike (PS) responses, as shown in the inserts in (**A**). The activation of adenosine A_1_ receptor by 2-chloroadenosine (CADO) caused a similar concentration-dependent inhibition in the two groups (**A**,**B**). The levels of endogenous adenosine within prefrontocortical excitatory synapses were lower upon caffeine treatment, as indirectly assessed by the disinhibition of synaptic transmission by the A_1_ receptor antagonist DPCPX (**C**,**D**). Long-term potentiation (LTP), triggered by a high-frequency train (HFS, 5 trains of 300 pulses at 100 Hz every 3 min), displayed a similar magnitude (quantified as the percentage change between the average amplitude of the 10 averaged potentials taken between 35 and 45 min after LTP induction in relation to the average PS amplitude measured during the 10 min that preceded LTP induction) in caffeine- and water-consuming mice and there was no modification of the effect on LTP of the selective antagonist of adenosine A_2A_ receptors, SCH58261 (SCH), between control and caffeine-treated mice (**E**,**F**). Data are mean ± SEM of 6–8 experiments (number of different animals tested); * *p* < 0.05 using Student’s *t* test with Welsh correction for comparison between two groups.

In synapses between projections of layer 2/3 and pyramidal neurons of layer V of slices from the prefrontal cortex (PFC), the activation of A_1_R by 2-chloroadenosine caused a similar concentration-dependent inhibition of synaptic transmission in mice drinking caffeine and in control mice (Figure 4A,B). However, the tonic activation of A_1_R by endogenous adenosine was lower in mice drinking caffeine than in control mice, as concluded from the lower (*p* < 0.05; Student’s *t* test) disinhibition of synaptic transmission by the selective A_1_R antagonist DPCPX (100 nM) in mice drinking caffeine than in control mice (Figure 4C,D). Finally, there was no significant difference (*p* = 0.879; Student’s *t* test) of the magnitude of LTP in PFC slices from mice drinking caffeine (71.8 ± 16.0% over baseline, n = 8) or water (62.7 ± 9.27% over baseline, n = 8) (Figure 4E,F) and there was no difference in the impact of the selective A_2A_R antagonist SCH58261 (50 nM) on LTP between both groups of mice (Figure 4F).

### 2.5. Impact of Caffeine Intake on Adenosine Modulation of Synaptic Transmission and Plasticity in the Amygdala

**Figure 5:**
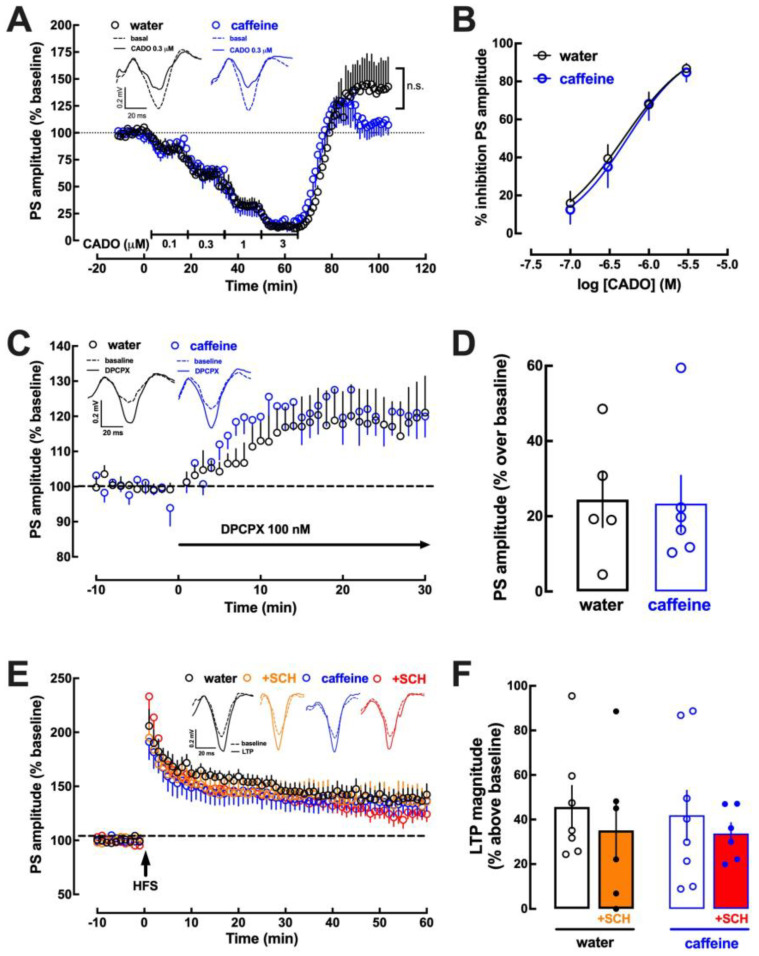
Impact of caffeine intake on the adenosine modulation of synaptic transmission and plasticity in the amygdala. Male adult mice (10 weeks old) consumed either caffeine (0.3 g/L, blue symbols and bars) or water (control, black symbols and bars) for 19–21 days before sacrifice. Synaptic transmission between the external capsule and the lateral amygdala was measured as the amplitude of the population spike (PS) responses, as shown in the inserts in (**A**). The activation of adenosine A_1_ receptor by 2-chloroadenosine (CADO) caused a similar concentration-dependent inhibition in the two groups (**A**,**B**). The levels of endogenous adenosine within amygdala excitatory synapses were similar between the two groups, as indirectly assessed by the disinhibition of synaptic transmission by the A_1_ receptor antagonist DPCPX (**C**,**D**). Long-term potentiation (LTP), triggered by a high-frequency train (HFS, 3 trains of pulses of 100 Hz delivered with a 5 s), displayed a similar magnitude (quantified as the percentage change between the average amplitude of the 10 averaged potentials taken between after LTP induction in relation to the average slope of PS amplitude measured during the 10 min that preceded LTP induction) in caffeine- and water-consuming mice and there was no modification of the effect on LTP of the selective antagonist of adenosine A_2A_ receptors, SCH58261 (SCH), between control and caffeine-treated mice (**E**,**F**). Data are mean ± SEM of 5–8 experiments (number of different animals tested).

In synapses between cortico-thalamic projections and the lateral amygdala, the activation of A_1_R by 2-chloroadenosine caused a similar concentration-dependent inhibition of synaptic transmission in mice drinking caffeine and in control mice (Figure 5A,B). There was also no modification of the tonic activation of A_1_R by endogenous adenosine as concluded from the similar (*p* = 0.931; Student’s *t* test) disinhibition of synaptic transmission by the selective A_1_R antagonist DPCPX (100 nM) in mice drinking caffeine and in control mice (Figure 5C,D). Finally, there was no significant difference (*p* = 0.613; Student’s *t* test) of the magnitude of LTP in amygdala slices from mice drinking caffeine (41.9 ± 11.2% over baseline, n = 8) or water (45.6 ± 9.56% over baseline, n = 7) (Figure 5E,F) and there was no difference in the impact of the selective A_2A_R antagonist SCH58261 (50 nM) on LTP between both groups of mice (Figure 5F).

### 2.6. Impact of Caffeine Intake on Metabolic Features of Cortical Synapses

**Figure 6:**
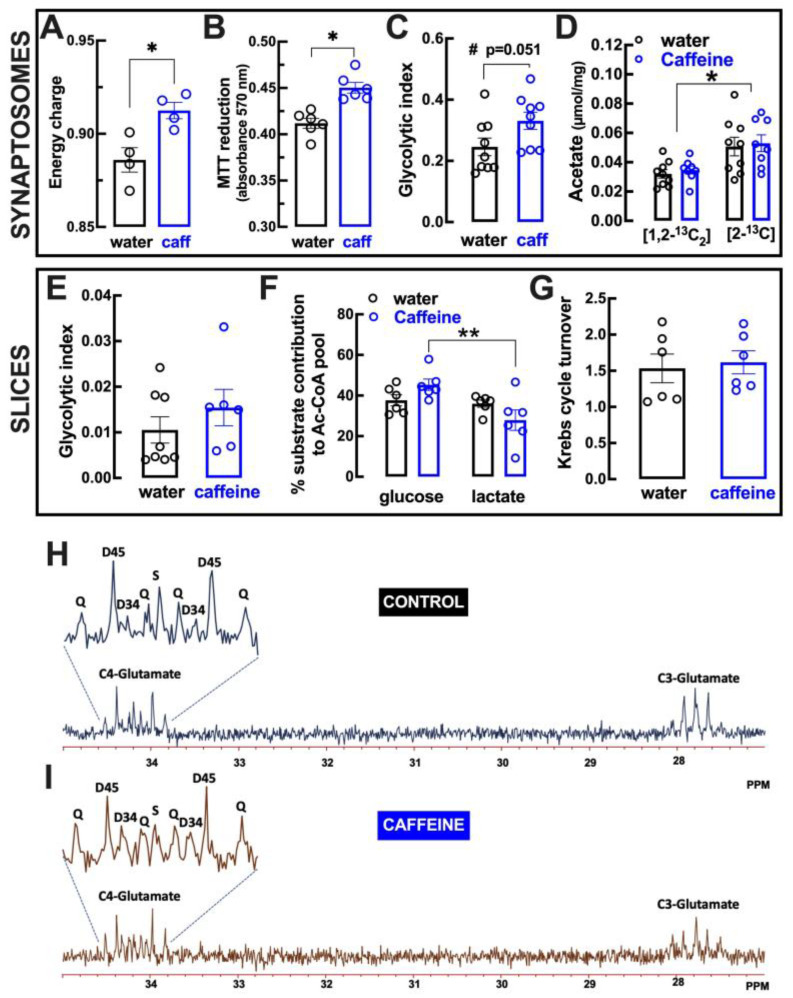
Impact of caffeine intake on primary metabolism in the cerebral cortex. Male adult mice (10 weeks old) consumed either caffeine (0.3 g/L, blue symbols and bars) or water (control, black symbols and bars) for 15 days before sacrifice and preparation of either cerebrocortical synaptosomes or slices (400 μm). Compared with control mice (10 weeks old) drinking water (black symbols and bars), cerebrocortical synaptosomes from mice consuming caffeine displayed a larger energy charge (**A**), assessed by HPLC quantification; a greater redox potential, assessed by a MTT reduction assay (**B**); a tendency for an increased glycolytic index, assessed by NMR quantification of the ratio between [U-^13^C]lactate production (in glucose equivalents) and [U-^13^C]glucose consumption (**C**); and a preserved production of acetate, assessed by NMR quantification of [1,2-^13^C_2_]acetate and [2-^13^C]acetate originating, respectively, from [U-^13^C]glucose and [3-^13^C]lactate (**D**). Data are mean ± SEM of 4-9 experiments (number of different animals tested); * *p* < 0.05 using Student’s *t* test with Welsh correction. Compared with control mice (black symbols and bars), cerebrocortical slices from mice consuming caffeine displayed a preserved glycolytic index (**E**), a reduced contribution of lactate for the pool of acetyl-CoA (**F**), and a preserved Krebs cycle turnover (**G**), which was estimated by the ratio of C4Q and C4D45 analysis of the glutamate C4 resonance and its multiplets, as illustrated in the representative NMR spectra (**H**,**I**). Data are mean ± SEM of 6–8 experiments (number of different animals tested); # *p* = 0.051 * *p* < 0.05 and ** *p* < 0.01 using Student’s *t* test.

Since omic studies identified adaptive changes of primary metabolism upon regular intake of caffeine [41], we next enquired if the regular intake of a moderate dose of caffeine would affect the metabolic properties of cortical synapses that are a major determinant of synaptic function and resilience under noxious conditions [55,56]. The study was carried out in synaptosomes from the mouse cerebral cortex, which is the largest brain region to provide sufficient material to complete the study. As shown in Figure 6A, there was an increased energy charge of synaptosomes (purified synapses) from mice drinking caffeine compared with control mice. Furthermore, there was an increased redox capacity, as assessed by the larger MTT reduction, in synaptosomes from mice drinking caffeine compared with control mice (Figure 6B). This was coupled with a tendency (*p* = 0.051; Student’s *t* test) towards a greater glycolytic index in synaptosomes from mice drinking caffeine compared with control mice (Figure 6C). In spite of this apparent increase in the glycolytic index, the analysis of the incorporation of ^13^C in acetate showed that synaptosomes from mice drinking caffeine preserved the same ability (*p* > 0.05; Student’s *t* test) of synaptosomes from control mice to preferentially metabolize lactate (increased levels of [2-^13^C]acetate originating from [3-^13^C]lactate) rather than using glycolytic products (lower levels of [1,2-^13^C2]acetate originating from [U-^13^C]glucose) (Figure 6D).

In cortical slices, there was no evident modification (*p* > 0.05; Student’s *t* test) of the glycolytic index in caffeine-drinking mice compared with control mice (Figure 6E), indicating that the putative increased glycolysis in caffeine-drinking mice occurred specifically in synapses. It was also observed that the contribution of lactate for the pool of acetyl-CoA was decreased in slices from caffeine-drinking mice compared with control mice (Figure 6F). Finally, ^13^C-isotopomeric analysis indicated that there was a similar turnover of the citrate (Krebs) cycle in cortical slices from control and caffeine-treated mice (Figure 6G).

## 3. Discussion

The regular intake of moderate doses of caffeine affords a robust neuroprotection that has been mainly associated with the ability of caffeine to antagonize adenosine receptors involved in the control of synaptic transmission, synaptic plasticity and synaptotoxicity [30]. Furthermore, a multi-omics study revealed that the regular consumption of caffeine promotes adaptive modifications in the hippocampus with signatures indicative of an altered primary metabolism and an altered efficiency of synaptic plasticity [41]. However, the present study refuted the hypothesis that caffeine intake may trigger a modification of synaptic transmission or synaptic plasticity in different brain regions (hippocampus, prefrontal cortex and amygdala), while demonstrating that these regions preserved a similar pattern of modulation by adenosine A_1_ and A_2A_ receptors irrespective of the regular exposure to caffeine. In contrast, we observed that the regular intake of caffeine altered cortical synaptic metabolism, bolstering the energy charge and the redox state of cortical synapses. This might be associated with a greater coupling of astrocytic and synaptic metabolism as indirectly inferred from the reduced usage of lactate by slices (predominantly extra-synaptic and astrocytic compartments) relieving a greater potential supplementation of lactate to support synaptic metabolism.

The behavioral analysis of mice regularly consuming a moderate dose of caffeine showed a global preservation of locomotion, mood and memory, largely in accordance with our previous similar reports in both mice (e.g., [19,27]) and rats (e.g., [15,18]). Accordingly, in keeping with the contention that synaptic plasticity is the neurophysiological basis of adaptive traits such as spatial memory or fear acquisition [63,64], we also observed that there was no major change of long-term potentiation (LTP) in different brain areas, namely, the hippocampus, prefrontal cortex or amygdala. These observations further support the previous conclusion that the regular consumption of caffeine does not trigger behavior or neurophysiological modifications but instead prevents modifications caused by deleterious factors such as sleep, fatigue or triggers of brain damage (reviewed in [29]). Importantly, the present study focused on behavior and neurophysiological alterations related to locomotion, mood and memory, and it still remains to be tested if the chronic consumption of caffeine might impact on other important behavioral outputs known to be controlled by caffeine such as circadian rhythm [65], sleep/arousal [66], addition [67], food intake [68], vascular control [69] or changes in the properties of the blood brain barrier [70].

Since moderate doses of caffeine selectively antagonize adenosine receptors [6,7] and the activity of the main adenosine receptors in the brain (A_1_R and A_2A_R; [49]) has a profound impact on brain dysfunction and neurodegeneration (reviewed in [30,71]), we were particularly surprised to conclude that the regular consumption of caffeine caused very mild modifications of the adenosine neuromodulation system in synapses of different brain areas. Although previous studies have reported that caffeine exposure alters the density of adenosine in the brain [8,43,44,72], only one study focused on alterations specifically of synaptic adenosine receptors [27], which are responsible for the most prominent effects of adenosine receptors in the brain, namely, the A_1_R-mediated control of synaptic transmission and dendritic membrane potential and the A_2A_R-mediated control of synaptic plasticity [30,49]. In accordance with this previous report of a lack of major alterations of the density of synaptic adenosine receptors [27], we now observed that the regular consumption of moderate amounts of caffeine does not seem to consistently alter the efficiency of adenosine receptors to control synaptic transmission and plasticity. In fact, we observed that caffeine intake triggered an increased efficiency of A_1_R to control synaptic transmission in the hippocampus but not in the prefrontal cortex or in the amygdala, whereas there was no consistent alteration of the ability of the A_2A_R antagonist to control LTP in any of the studied brain regions. The only more consistent alteration upon regular caffeine intake was the observation of decreased basal levels of adenosine controlling synaptic transmission in the hippocampus and prefrontal cortex, but not in the amygdala. Although we have not characterized putative alterations upon chronic exposure to caffeine of the expression or density of adenosine receptors in different cell types or subcellular compartments in the different brain areas, the present functional data suggest that the impact of caffeine on the extracellular levels of adenosine might be opposite in the brain and in the plasma, where it was previously reported that caffeine exposure increased the plasma levels of adenosine [42].

This global conclusion that neither synaptic function nor the modulation of synaptic function by adenosine are altered upon regular consumption of caffeine is somewhat surprising since synaptic dysfunction is consistently observed at the onset of most brain diseases that are prevented by caffeine intake, such as epilepsy [73,74], depression [27,45], Alzheimer’s [9,75] or Parkinson’s disease [76,77]. Thus, it would be expected that caffeine might bolster synaptic function to increase their resilience to degeneration under noxious brain conditions. Indeed, we observed that cortical synapses displayed a more robust energy and redox status upon exposure to caffeine, although this more robust metabolic state of synapses did not translate into an altered synaptic function, since the metabolic status of control synapses is likely fully sufficient to sustain the synaptic function imposed experimentally. However, since metabolic deficits are present in brain diseases and trigger synaptic dysfunction [55,56], it is expected that a more robust energy charge and redox state will bolster the sustainability of synapses under noxious brain conditions. Therefore, this increase in the metabolic robustness of cortical synapses provides a tentative synapse-centered explanation for the neuroprotection afforded by chronic caffeine intake, although it still remains to be determined if caffeine triggers similar metabolic alterations in synapses of the hippocampus or amygdala.

However, our efforts to unveil the putative alterations of primary metabolism that underlie this caffeine-induced increase in the metabolic robustness of cortical synapses did not provide a straightforward answer. In fact, although we observed that the exposure of cortical synapses to caffeine seemed to increase the glycolytic index, we did not find an increased contribution of glycolysis for the pool of acetylCoA in cortical synapses. This might be due to the fact that lactate is a greater contributor than glycolytic products for the formation of acetate in nerve terminals, irrespective of the exposure to caffeine. In parallel, we observed that caffeine exposure decreased the utilization of lactate as a source of aceyl-CoA in slices, where synapses only account for circa 2% of volume, implying that metabolic features are mostly representative of extra-synaptic compartments, in particular of astrocytes. In keeping with the importance of the astrocytic shuttling of lactate to fuel synaptic activity [78], the presently observed simultaneous maintenance of the glycolytic index and decreased contribution of lactate to the pool of acetyl-CoA in slices is suggestive of a greater availability of lactate to be shuttled and used in synapses. Thus, the repeated exposure to caffeine would simultaneously increase glycolytic flow rate as well as the availability of lactate to bolster the metabolic robustness of cortical synapses. This scenario underscores the importance of the altered function of different compartments to understand how the intake of caffeine predisposes the brain to better handle noxious stimuli. In fact, apart from the metabolic adaptation of cortical synapses, astrocytes also emerge as a putative target to be modified by caffeine, as hinted by previous observations (e.g., [79,80]) but still awaiting a direct demonstration. In this respect, it is important to keep in mind that the neuroprotection afforded by caffeine might also involve additional cell types such as microglia orchestrating neuroinflammation and neuro-vascular coupling that also contribute to the outcome of brain damage (reviewed in [81,82]) and that are controlled by caffeine [69,83]. The putative participation of different brain cell types and compartments in the adaptive effects of caffeine that are responsible for the neuroprotection afforded by caffeine also raises the question of better understanding how caffeine actually distributes throughout different brain compartments. This is particularly relevant in view of the observed increase in the levels of caffeine in the plasma and in the brain parenchyma from an acute to a chronic exposure to caffeine, implying that a different pharmacokinetic profile of caffeine might emerge upon continuous exposure to caffeine, a factor that may be decisive to clearly distinguish the effects of acute versus chronic exposure to caffeine.

Some limitations should be kept in mind in relation to the presently concluded metabolic rather than behavioral or neurophysiological effects resulting from chronic caffeine intake. First, as previously mentioned, we focused on behavioral effects related to locomotion, mood and memory, and several other relevant behavioral outputs were not assessed in the present study, such as alterations of circadian rhythms, sleep, addiction or hemodynamic effects. Additionally, the study was carried out in male adult mice, and there is previous evidence of sex-differentiated effects of caffeine [84,85]. Additionally, the extrapolation of these findings to humans requires some caution in view of the different pharmacokinetics of caffeine in different species [86], the different brain organization, and different lifestyles that affect the impact of caffeine on rodents and humans. 

## 4. Materials and Methods

### 4.1. Animals

Male C57bl\6j mice (10–12 weeks old; total of 36) were from Charles River (Barcelona, Spain). Animals were maintained in groups of two or four per cage in a temperature-controlled room (22 ± 2 °C), with free access to food and water, and with a 12 h light/12 h dark cycle (lights on at 7:00 am). The study was performed in accordance with the principles and procedures outlined as “3Rs” in the guidelines of the European Union (2010/63/EU), FELASA and ARRIVE and was approved by the Ethics Committee for Animal Research of the Center for Neuroscience and Cell Biology of the University of Coimbra (ORBEA 238-2019/14102019). All efforts were made to reduce the number of animals used and to minimize their stress and discomfort. In all manipulations, the experimenters were unaware of the experimental group to which each animal belonged. 

### 4.2. Drugs and Treatments

Caffeine (from Sigma) was prepared in a 0.3 g/L solution in water provided *ad libitum* to half the mice during at least 2 weeks starting at 10 weeks of age until sacrifice, and compared with a control group of mice provided only with water. This dose of caffeine p.o. has previously been shown to afford neuroprotection against different noxious brain stimuli (e.g., [27,87,88]) and yields a plasma concentration of caffeine roughly equivalent to that obtained in humans consuming an average of 3–4 cups of coffee daily [6]. Caffeine solutions were kept in dark bottles for protection from light and were changed every 5 days. Mice had free access to caffeine until their sacrifice.

2-Chloroadenosine (CADO) and 8-cyclopentyl-1,3-dipropylxanthine (DPCPX) were from Sigma, and 7-(2-phenylethyl)-5-amino-2-(2-furyl)-pyrazolo-[4,3-e]-1,2,4-triazolo[1,5-c]pyrimidine (SCH58261) was from Tocris. Stock solutions of either DPCPX (5 mM) or SCH58261 (5 mM) were prepared in dimethylsulfoxide (Sigma) and a dilution was prepared in superfusion medium, controlling for the impact of the residual amount of dimethylsulfoxide. CADO preparation was performed in water to a stock solution of 10 mM. CADO was applied in four increasing concentrations: 0.1, 0.3, 1, and 3 μM, as previously conducted to estimate the potency and efficacy of A_1_R [60]. DPCPX (100 nM) and SCH58261 (50 nM) were both used at supramaximal but selective concentrations [61,62]. All other chemical substances used, unless stated otherwise, were from Sigma.

### 4.3. Behavioral Analysis 

We used a tight schedule of behavioral characterization, with a minimal time interval between each test to avoid cross-interference between the tests [27,88]. All behavior tests were carried out from 9 a.m. to 4 p.m. in the 16th until the 18th days after beginning the free intake of caffeine, by experimenters who were unaware of drug treatments, in a sound-attenuated room with an 8 lux illumination and visual cues on the walls, to which the animals were previously habituated. The apparatuses were cleaned with 10% ethyl alcohol to remove any odors after testing each animal.

Locomotion and exploratory behavior were monitored in the morning of day 16, using an open-field arena, where each mouse was placed in the center of the open field to record during 10 min the following variables: number of peripheral squares (adjacent to the walls) crossed (peripheral locomotion), number of central squares (away from the walls) crossed (central locomotion), and total locomotion (peripheral locomotion plus central locomotion).

Hippocampal-dependent memory was evaluated using the object displacement test, carried out in the afternoon of day 16. Mice were exposed to two identical objects in the same open field apparatus in which they were habituated and were allowed to explore for 8 min the objects fixed in opposite corners 5 cm away from walls and 25 cm apart from each other. In the test trial, carried out 2 h later, mice were again placed for 5 min in the open field arena, except that one of the objects was moved to a novel position. Memory performance was quantified with an object displacement index defined as the ratio between the time exploring the object in the novel location over the total time exploring both objects. Exploration of an object was defined as directing the nose to the object at a distance equal to or less than 1 cm from the object and/or touching it with the nose; rearing on to object was not considered exploratory behavior.

Spatial memory was further evaluated using a 2-trials Y-maze paradigm [89] on the morning of day 17. The test consisted of two sessions of 8 min duration separated by a 2 h inter-trial interval. During the first session, the mouse was placed at the end of one arm and allowed to explore the two available arms since the third arm (the novel arm) was blocked by a guillotine door. During the second session, the ‘novel’ arm was open, the mouse was placed in the start arm and allowed to explore the three arms. Memory performance was evaluated by measuring the time spent exploring the ‘novel’ arm compared with the exploration of the other two arms. An entry into an arm was defined as placement of all four paws into the arm.

Anxiety was further assessed in the afternoon of day 17, using the elevated-plus maze, where each animal was allowed to explore the maze for 8 min. The number of entries and the time spent in both open and closed arms were recorded, considering an entry only when the whole body and four paws were inside an arm. 

Depressive-like behavior was evaluated in the forced swimming test, carried out in the morning of day 18. Mice were placed in individual glass cylinders (40 cm in height and 17 cm in diameter) containing water (water depth was 30 cm, kept at 25 ± 1 °C) to measure the total duration of immobility, climbing and swimming during a 10 min session. A mouse was regarded as immobile when floating motionless or making only those movements necessary to keep its head above the water. The climbing behavior was defined as upward-directed movements of the forepaws usually along the side of the swimming chamber and the swimming behavior was defined as movement (usually horizontal) throughout the swimming chamber; diving and face shaking behaviors were not considered.

Mice were sacrificed in pairs (1 control and 1 caffeine-treated) between days 19 and 22 after the start of treatment by decapitation after deep halothane anesthesia.

### 4.4. Caffeine Quantification 

A blood sample (200 μL) was collected at the time of sacrifice of the mice and the serum was separated by centrifugation and stored for quantification of caffeine and its xanthine metabolites, as previously described [19], by HPLC using a reverse-phase column (LiChro-CART 125 × 4 mm LiChrospher 100 RP-18 (5 μm) cartridge fitted into a ManuCART holder (Merck)) and a Gilson system equipped with a UV detector set at 274 nm. The eluent was 40% (*v*/*v*) methanol, pH 6.0, with a flow rate of 0.8 mL/min. Identification and quantification of caffeine and its demethylated products (theophylline, theobromine, paraxanthine) were performed by comparison of their relative retention time with standards (0.1–100 μΜ). 

Additionally, different brain areas were dissected from one hemisphere, namely, the cerebral cortex, the hippocampus and the striatum. After weighting for normalization, each of these samples was homogenized in 2 mL of methanol-acetone (4:1), mixed for 15 min, centrifuged at 3000× *g* for 15 min, and 20 μL of the supernatant were used for HPLC analysis of caffeine. The concentration of caffeine was calculated based on the estimated volumes of C57bl\6j young adult mouse brain areas, namely, 109 mm^3^ for the cerebral cortex, 22 mm^3^ for the hippocampus, and 15 mm^3^ for the striatum [90]. Note that this estimate assumed a homogenous distribution of caffeine in each brain sample. 

### 4.5. Slice Electrophysiology 

After sacrifice, the brain was quickly placed in ice-cold, oxygenated (95% O_2_, 5% CO_2_) artificial cerebrospinal fluid (ACSF; in mM: 124.0 NaCl, 4.4 KCl, 1.0 Na_2_HPO_4_, 25.0 NaHCO_3_, 2.0 CaCl_2_, 1.0 MgCl_2_, 10.0 glucose). Using a Leica VT1200S vibratome, dorsal hippocampal slices (400 µm thick), coronal slices containing the prelimbic medial prefrontal cortex (PFC, 300 μm thick) and horizontal slices containing the amygdala (400 µm thick) were cut and placed in a holding chamber with oxygenated ACSF. Slices were allowed to recover at 32–34 °C for at least 1 h prior to recording, when they were transferred to a submerged recording chamber and superfused at 3 mL/min with oxygenated ACSF kept at 30.8 °C. 

The configuration of the extracellular recordings was as previously described: in the hippocampus, the stimulating bipolar concentric electrode was placed in the proximal CA1 stratum radiatum for stimulation of the Schaffer collateral fibers and the recording electrode, filled with 4 M NaCl (2–5 MΩ resistance), was placed in the CA1 stratum radiatum targeting the distal dendrites of pyramidal neurons [7,91]; in the amygdala, the stimulating electrode was placed in the external capsule and the recording electrode was placed in the lateral nuclei of the amygdala [92]; in the prefrontal cortex, the stimulation electrode was placed in layer II/III and the recording electrode was placed in layer V [93]. Stimulation was performed using either a Grass S44 or a Grass S48 square pulse stimulator (Grass Technologies) or a Digitimer DS3 stimulator (Digitimer LTD), with rectangular pulses of 0.1 ms applied every 15–20 s. After amplification (ISO-80, World Precision Instruments, U.K.; or AxoPatch 200B amplifier, Axon Instruments, U.S.A.), the recordings were digitized (PCI-6221 acquisition board, National Instruments or Digidata 1322A, Axon Instruments), averaged in groups of 3-4, and analyzed using either the ClampFit version 10.5 program (Axon Instruments) or the WinLTP version 2.10 software [94]. The intensity of stimulation was chosen to be between 40 and 50% of maximal field excitatory postsynaptic potential (fEPSP; in the hippocampus) or population spike (PS) response (in the amygdala and prefrontal cortex), based on input/output curves in which the percentage of maximum fEPSP slope or PS amplitude was plotted versus stimulus intensity. 

Alterations of synaptic transmission were quantified as the % modification of the average value of the fEPSP slope or the PS amplitude taken from 10 to 15 min after beginning the application of different CADO concentrations, in relation to the average value of the fEPSP slope or PS amplitude during the 5 min that preceded the application of each new concentration. For DPCPX, we compared fEPSP slope or PS amplitude values in the 5 min that preceded the application of the drug in relation to the average values of the 15 to 20 min after its exposure.

Long-term potentiation (LTP) was induced by high-frequency stimulation (100 Hz for 1 s in hippocampal synapses; 5 trains of pulses of 3 s duration at 100 Hz, delivered with a 3 min interval in prefrontocortical synapses; 3 trains of pulses of 1 s duration at 100 Hz delivered with a 5 s interval in amygdala synapses). LTP was quantified as the percentage change between two values: the average slope or amplitude of the 10 averaged potentials taken after LTP induction (between 50 and 60 min in the hippocampus and amygdala, and between 35 and 45 min in the prefrontal cortex) in relation to the average slope of the fEPSP or the PS amplitude measured during the 10 min that preceded LTP induction. The effect of caffeine on LTP was assessed by comparing LTP amplitude in slices from untreated versus treated animals.

### 4.6. Energy Charge, Redox Potential and Primary Metabolism in Synaptosomes

In order to analyze metabolic features in cerebrocortical synapses, we took advantage of synaptosomes, essentially as described and validated previously [95]. The preparation began with the homogenization of fresh brain cortical tissue in an isotonic sucrose-HEPES solution at 4 °C and pH 7.4, allowing the separation between nerve terminal and respective axon, followed by two differential centrifugations. After measuring the protein content (BCA Protein Assay Kit, Pierce, Thermo Scientific), the synaptosomes were suspended in Krebs–HEPES medium (composition in mM: 125 NaCl, 3 KCl, 1.25 NaH_2_PO_4_, 1 MgCl_2_, 2 CaCl_2_, 10 mM glucose, 25 HEPES; pH 7.4) and maintained on ice until use. 

To determine the energy charge of synaptosomes, defined as ([ATP] + ½ [ADP])/([ATP] + [ADP] + [AMP]), the synaptosomes (120–180 μg of protein) were equilibrated at 37 °C for 20 min in 200 μL of Krebs–HEPES medium. The mixture was then frozen with liquid nitrogen, homogenized in 3 M perchloric acid, centrifuged and the supernatant neutralized with 4 M NaOH with 0.4 M Tris base, as previously described [96]. Adenine nucleotides and adenosine were separated and quantified in the neutralized extract by reverse-phase HPLC [97] using 100 μM ITP as internal standard to correct for recovery in the extraction procedure, as previously described [96]. 

The redox status of synaptosomes was measured by a colorimetric assay using 3-(4,5-dimethylthiazol-2-yl)-2,5-diphenyltetrazolium bromide (MTT) (Sigma), as previously described [98]. Synaptosomes (120–180 μg of protein) were incubated for 20 min at 37 °C in 200 μL of Krebs–HEPES medium and MTT (0.5 mg/mL) was then added and incubated for 1 h at 37 °C in the dark. As MTT is converted to a water-insoluble blue product (formazan) by synaptosomes, the precipitated dye can be spectrophotometrically (570 nm) quantified after exposing synaptosomes to isopropanol containing 0.04 M HCl. 

To assess primary metabolism, synaptosomes were incubated at 37 °C for 7 h in carbogenated (95% O_2_ + 5% CO_2_) Locke’s buffer (composition in mM: 154 NaCl, 5.6 KCl, 2.3 CaCl_2_, 1.0 MgCl_2_, 3.6 NaHCO_3_ and 5.0 HEPES; pH 7.4), to obtain a metabolic steady-state, in the presence of ^13^C labelled substrates, namely, 5 mM [U-^13^C]glucose (Cambridge Isotopes Laboratories) and 2 mM [3-^13^C]lactate (Cambridge Isotopes Laboratories). After the 7 h incubation, the synaptosomes and medium were separated through centrifugation (16,000× *g* for 2 min at 4 °C), and samples were prepared for analysis by NMR spectroscopy, as described below. 

### 4.7. Superfusion of Cortical Slices

The analysis of primary metabolism in slices was carried out as previously described in detail [99]. Briefly, coronal cortical slices (400 μm) were cut with a Mcllwain tissue chopper, placed in a submerged chamber and superfused (3 mL/min) for 60 min in a close-loop circuit with carbogenated (95% O_2_ + 5% CO_2_) modified Krebs solution (composition in mM: 115 NaCl, 3.0 KCl, 2.0 CaCl_2_, 1.2 MgSO_4_, 1.2 KH_2_PO_4_, 25 NaHCO_3_; pH 7.4), kept at 37 °C, to allow their stabilization. The ^13^C-labelled substrates, namely, 5 mM [U-^13^C]glucose and 2 mM [3-^13^C]lactate, were then added through the superfusion solution and left for 7 h, to reach metabolic steady-state [99]. Extracellular medium samples were collected (180 μL), 45 μL of sodium fumarate (10 mM) D_2_O (99.9%) solution was added as internal standard, and the samples were stored at -20 °C for analysis by ^1^H-NMR spectroscopy. To obtain the aqueous extracts of cortical slices, they were transferred to a previously frozen porcelain grinder (−80 °C) and 300 μL of 7% (*v/v*) perchloric acid (PCA) was added. The grinded tissue mixed with PCA was centrifuged (16,000× *g* for 15 min at 4 °C) and the supernatant, containing the water-soluble metabolites, was neutralized with a decreasing gradient of KOH concentrations (from 10 M to 0.01 M). The aqueous extracts were centrifuged to remove the salt formed (KClO_4_) with the addition of KOH, lyophilized, and stored in an exicator until ^13^C-NMR spectra acquisition.

### 4.8. Nuclear Magnetic Resonance (NMR) Spectroscopy

Primary metabolism was assessed using ^1^H- and ^13^C-nuclear magnetic resonance (NMR), as previously described [99]. NMR spectra were acquired at the NMR facility CERMAX in ITQB-NOVA using a Bruker 500 MHz Ultrashield Neo NMR spectrometer using a 5 mm TCI C/N Prodigy Cryo probe at 25 °C. The ^1^H-NMR spectra were obtained using a 30° pulse, 5882 kHz spectral width, 65,536 number of points, and 10 s of interpulse recycling time (3 s of acquisition time and 7 s pulse delay), whereas the ^13^C-NMR spectra were obtained using 30° pulse, 30,120 Hz spectral width, 32,768 number of points, and 2 s of interpulse recycling time (1.5 s of acquisition time and 0.5 s pulse delay). For the quantification of metabolite levels present in medium samples and aqueous extracts, sodium fumarate was used as an internal standard. 

For the ^1^H-NMR analysis of medium samples, the following metabolic parameters were evaluated: (i) concentration of [1,2-^13^C2]acetate originating from [U-^13^C]glucose, and of [2-^13^C]acetate originating from lactate [3-^13^C]lactate, measured around 1.9–2.0 ppm; (ii) consumption of [U-^13^C]glucose and [3-^13^C]lactate consumption, measured around 5.4 ppm and 1.4–1.5 ppm, respectively; (iii) the glycolytic index, calculated as the ratio between [U-^13^C]lactate production (in glucose equivalents) and [U-^13^C]glucose consumption. 

For the ^13^C NMR analysis of extract samples from slices, we quantified the glutamate C4 multiplet resonances (C4Q, C4D45, C4D34 and C4S) by deconvolution. The contribution of [U-^13^C]glucose versus [3-^13^C]lactate for acetyl-CoA enrichment was estimated by the ratio between C4Q and C4D34, which represented pure contributions from the two ^13^C-labeled substrates. Citric (Krebs) cycle turnover was estimated by the ratio of C4Q and C4D45.

### 4.9. Statistical Analyses

The values presented are mean ± S.E.M. with the number of determinations (n, preparations from different mice). The comparison of two experimental conditions was performed using a two-tail Student’s *t* test with Welsh correction. Otherwise, statistical analysis was performed by two-way analysis of variance (ANOVA), followed by a Newman–Keuls post hoc test. *p* < 0.05 was considered to represent statistical significance. Statistical analysis was performed using GraphPad Prism software (GraphPad Software, CA, USA).

## 5. Conclusions

It was concluded that caffeine intake does not trigger evident alterations of behavior or of synaptic plasticity; however, caffeine intake increases the metabolic competence of synapses, which might tentatively be related with the previously described better ability of animals consuming caffeine to cope better with deleterious stimuli triggering brain dysfunction [29].

## Data Availability

The data that support the findings of this study are available from the corresponding author upon reasonable request.

## Abbreviations

A1R	adenosine A1 receptors
A2AR	adenosine A2A receptors
ACSF	artificial cerebrospinal fluid
CADO	2-chloroadenosine
DPCPX	1,3-dipropyl-8-cyclopentylxanthine
EPSP	excitatory post-synaptic potential
LTP	long-term potentiation
MTT	3-(4,5-dimethylthiazol-2-yl)-2,5-diphenyltetrazolium bromide
NMR	nuclear magnetic resonance
PCA	perchloric acid
PS	population spike
RT	room temperature
SCH58261	2-(2-furanyl)-7-(2-phenylethyl)-7H-pyrazolo[4,3-e][1,2,4]triazolo[1,5-c]pyrimidin-5-amine
